# Development and validation of a nomogram for survival prediction in hepatocellular carcinoma after partial hepatectomy

**DOI:** 10.1186/s12893-023-01922-x

**Published:** 2023-01-30

**Authors:** Yang Lu, Shuang Ren, Jianning Jiang

**Affiliations:** 1 Department of Infectious Diseases, The First Affiliated Hospital of Guangxi Medical University, Nanning, 530021 Guangxi China; 2 Department of Comprehensive Internal Medicine, The Affiliated Tumor Hospital of Guangxi Medical University, Nanning, China

**Keywords:** Prognosis, Model, Surveillance, Hepatocellular carcinoma

## Abstract

**Background:**

The prognosis for hepatocellular carcinoma (HCC) is complex due to its high level of heterogeneity, even after radical resection. This study was designed to develop and validate a prognostic nomogram for predicting the postoperative prognosis for HCC patients following partial hepatectomy.

**Patients and methods:**

We extracted data on HCC patients and randomly divided them into two groups (primary and validation cohorts), using the Surveillance, Epidemiology and End Results (SEER) database. We developed the prediction model based on the data of the primary cohort and prognostic factors were evaluated using univariate and multivariate Cox regression analysis. A nomogram was constructed for predicting the 1-, 3-, and 5-year survival probability of HCC patients after surgery based on the results of the multivariate Cox regression analysis. The performance of the nomogram was evaluated in terms of its discrimination and calibration. To validated the model, discrimination and calibration were also evaluated in the validation cohort. Decision curve analysis (DCA) was performed to assess the clinical utility of the nomogram.

**Results:**

A total of 890 patients who underwent partial hepatectomy for HCC were included in the study. The primary cohort enrolled 628 patients with a median follow-up time of 39 months, the 1-, 3-, and 5-year survival rate were 95.4%, 52.7% and 25.8% during follow-up. Multivariate Cox regression analysis showed that differentiation, tumor size, AFP and fibrosis were independently association with the prognosis of HCC patients after partial hepatectomy. The nomogram showed a moderate discrimination ith a C-index of 0.705 (95% CI 0.669 to 0.742), and good calibration. Similar discrimination with a C-index of 0.681 (95% CI 0.625 to 0.737), and calibration were also observed in the validation cohort. Decision curve analysis showed that the nomogram could be useful to predicting the prognosis in HCC patients following partial hepatectomy.

**Conclusions:**

The proposed nomogram is highly predictive and has moderate calibration and discrimination, potentially contributing to the process of managing HCC patients after partial hepatectomy in an individualized way.

## Introduction

Primary liver cancer is one of the most common malignant tumors in the world, while hepatocellular carcinoma is the main type of primary liver cancer, accounting for 75% to 85%, leading cause of cancer deaths globally [1–3]. Since the beginning of the era of immunotherapy and targeted therapy, targeted therapy and immune checkpoint inhibitors have shown great prospects in the treatment of HCC, improving survival rate. However, there is a wide range of prognoses following surgical resection of early-stage HCC [4, 5]. Although a number of clinicopathological factors are associated with survival, accurate prognosis for HCC patients after surgery remains a challenge [6, 7].

A reliable prediction of HCC after surgery is not only essential for physicians and patients to make decisions about adjuvant treatment, type of treatment, and frequency of follow-up, but can also provide patients and their families with useful information about treatment modalities and outcomes. The Barcelona Clinic Liver Cancer (BCLC) staging system and The American Joint Committee on Cancer (AJCC) staging schema are the most widely used standard for staging HCC patients [8, 9]. Although BCLC or AJCC staging may be useful in predicting overall survival, risk stratification systems used to predict the prognosis of individual patients may be vary widely.

Minjun et al. [10] reported on the use of a nomogram to predict clinical outcomes among patients with HCC based on three medical centers’ data. Since the nomogram was developed using the results of the patient’s post-operative laboratory tests, may increase the financial burden on patients, so, the availability of their nomogram is limited. Other studies have attempted to construct models to assess the prognosis of HCC after surgical resection, but these studies are mostly small-sample, single-center reports [11–15]. Therefore, this study aimed to develop and validate a prognostic nomogram for predicting postoperative survival of patients with HCC based on the SEER database, which contains information from a large population of multiple centers.

## Patients and methods

### Ethics approval and consent of participate

Neither ethics approval nor patients informed consent for this study were required, as the data used is publicly available and do not contained personally identifiable information.

### Patients

This retrospective, observational cohort study was based on the SEER database, a cancer registry. Incidence-SEER 18 Regs Custom Data (with additional treatment fields), Nov 2018 Sub (1975–2016 varying) is the largest data source in the SEER database, which including approximately 34.6 percent of the population of the United States [16]. The SEER database routinely contains demographic, pathological, survival and follow‐up information, such as race, age, sex, degree of tumor differentiation, AJCC staging, pathologic type of tumor, radiotherapy or chemotherapy record, the operation information, and survival time, etc.

**Fig. 1:**
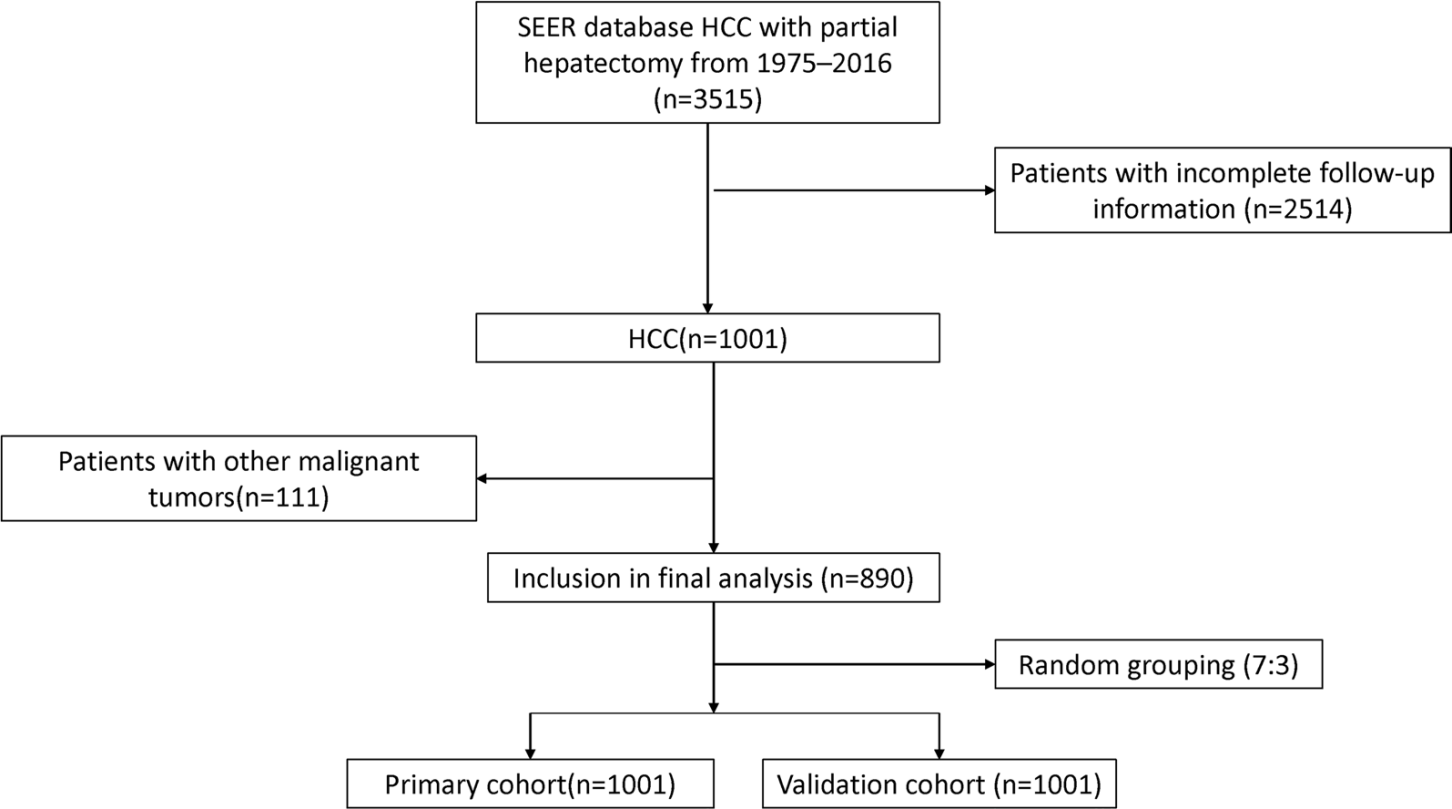
Numbers of patients enrolled in the primary cohort and validation cohort

Inclusion criteria were: (1) Patients with HCC diagnosed by liver pathology according to the ICD-O-3 histology codes, (2) The patient was treated with partial hepatectomy, (3) The follow-up time is greater than or equal to 1 month. Exclusion criteria were: (1) Patients with incomplete follow-up information, (2) Patients with other malignant tumors. Finally, 890 patients were included and analyzed in our study. All patients were randomly assigned to primary cohort (n = 628) and validation (n = 262) cohort in a 7:3 ratio. The selection and deletion process of patients is shown in Fig. 1.

### Data collection

The following information was obtained directly from SEER database: race, age, sex, the extent of tumor, AJCC staging (the seventh edition), T staging, N staging, M staging, tumor size, tumor differentiation, AFP, fibrosis, chemotherapy, radiation, primary site surgical information, survival status, and survival time. Information on distant metastases (such as lung, brain, bone, etc.) was only recorded in the SEER database after 2010 years. Therefore, we used SEER*Stat software 8.3.9 to extract the follow-up data of HCC patients from 2010 to 2016 years.

### Statistical analysis

All statistical analyses was performed with SPSS version 26.0 (IBM Corp, Armonk, NY, USA) and R software (rms [17] and survival [18] packages of R version 3.6.2 1; http://www.Rproject.org). The level of statistical significance was set to 0.05, and all significance tests were conducted using two-sided. Median [M (P25, P75)] represent continuous variables. Number and percentage represent categorical variables. The postoperative survival rate and median follow-up time were analyzed by Kaplan Meier and reverse Kaplan Meier method, respectively.

We evaluated the independent prognostic factors for HCC using univariate and multivariate Cox regression analysis. For exploring and controlling confounding factors, Spearman correlation was analyzed before multivariate Cox regression analysis. As described previously [19], a nomogram for predicting the 1-, 3-, and 5-year survival rates of HCC patients after surgery was constructed and adjusted based on the results of multivariate Cox regression. In each cohort, the performance of the nomogram was evaluated using the discrimination and calibration. The C-index was used to measure the degree of discrimination, ranging from 0.5 (not discriminating) to 1 (perfect discriminating) [20]. A calibration curve was plotted to evaluate the calibration ability of the nomogram. Using a decision curve analysis (DCA), we quantified the net benefits at various threshold probabilities in order to test for potential clinical benefit from the estimation model.

## Results

### Characteristics of patients in the primary and validation cohorts

The demographic and pathological data of 890 hepatocellular carcinoma patients after partial hepatectomy were analyzed. The primary and Validation cohort included 628 and 262 patients, respectively. Patients’ demographic and pathological characteristics were summarized in Table 1. There were no significant differences in baseline patient’ characteristics between the two cohorts.

**Table 1:** The demographic and clinicopathological characteristics of the primary and validation cohorts

Variable	Primary cohort	Validation cohort	P-value
Age, years	64 (58.70)	63 (57.70)	0.683
Race, n (%)			0.952
White	342 (54.4%)	146 (55.5%)	
Black	84 (13.4%)	34 (12.9%)	
Other	202 (32.2%)	83 (31.6%)	
Sex, n (%)			
Female	164 (26.2%)	76 (28.9%)	0.400
Male	463 (73.8%)	187 (71.1%)	
Differentiation, n (%)			0.944
Well	126 (20.1%)	55 (20.9%)	
Moderately	353 (56.3%)	145 (55.1%)	
Poorly/undifferentiated	148 (23.6%)	63 (24.0%)	
The extent of tumor, n (%)			0.546
Localized	506 (80.7%)	213 (81.0%)	
Regional	101 (16.1%)	45 (17.1%)	
Distant	20 (3.2%)	5 (1.9%)	
AJCC staging, n (%)			
I	345 (55.0%)	153 (58.2%)	0.471
II	164 (26.2%)	62 (23.6%)	
III	96 (15.3%)	43 (16.3%)	
IV	22 (3.5%)	5 (1.9%)	
T staging, n (%)			
T1	348 (55.5%)	157 (59.7%)	0.559
T2	172 (27.4%)	62 (23.6%)	
T3	85 (13.6%)	37 (14.1%)	
T4	22 (3.5%)	7 (2.7%)	
N staging, n (%)			0.751
N0	619 (98.7%)	261 (99.2%)	
N1	8 (1.3%)	2 (0.8%)	
M staging, n (%)			
M0	611 (97.4%)	260 (98.9%)	0.282
M1	16 (2.6%)	3 (1.1%)	
Tumor size, n (%)			
< 3 cm	138 (22.0%)	60 (22.8%)	0.988
3–5 cm	198 (31.6%)	84 (31.9%)	
5–10 cm	195 (31.1%)	79 (30.0%)	
≥ 10	96 (15.3%)	40 (15.2%)	
AFP, n (%)			
Positive	386 (61.6%)	160 (60.8%)	0.839
Normal	241 (38.4%)	103 (39.2%)	
Fibrosis, n (%)			
Normal	327 (52.2%)	142 (54.0%)	0.616
Cirrhosis	300 (47.8%)	121 (46.0%)	
MVI, n (%)			
Yes	142 (22.6%)	51 (19.4%)	0.282
No	485 (77.4%)	212 (80.6%)	
Radiotherapy, n (%)			
Yes	26 (4.1%)	8 (3.0%)	0.433
No	601 (95.9%)	255 (97.0%)	
Chemotherapy, n (%)			
Yes	88 (14.0%)	37 (14.1%)	0.990
No	539 (86.0%)	226 (85.9%)	

Other race: American Indian/AK Native, Asian/Pacific Islander

*AFP* the highest AFP test results documented in the medical record prior to treatment, *MVI* microvascular invasion

Patients in the primary cohort had a median follow-up of 39 months, and mean survival rate was 51 months, the 1-, 3-, and 5-year survival rate were 95.4%, 52.7% and 25.8%, whereas the median follow-up time in the validation cohort was 42 months and mean survival rate was 58 months, the 1-, 3-, and 5-year survival rate was 95.5%, 57.5% and24.0%. In the primary cohort, 246 (39.2%) patients died, and in the validation cohort, 80 (30.4%) patients died.

### Construction of the nomogram

The results of univariate and multivariable Cox proportional hazards regression analysis are show in Table 2. When confounding variables were adjusted, multivariate cox regression analysis demonstrated the following 4 variables were independently association with the prognosis of hepatocellular carcinoma patients after partial hepatectomy: Differentiation, Tumor size, AFP, and Fibrosis. The poorer differentiation, larger tumor size*,* positive of AFP, and cirrhosis were associated with a poorer prognosis.

**Table 2:** Cox PHs analysis showing the association of variables with prognosis in the primary cohort

Variable	Univariate analysis HR (95% CI)	P value	Multivariate analysis HR (95% CI)	P value
Age	0.943 (0.821–1.084)	0.412		
Race				
White	Reference			
Black	1.347 (0.959–1.892)	0.086		
Other	0.791 (0.591–1.059)	0.115		
Sex				
Male	Reference			
Female	1.329 (0.987–1.790)	0.061		
Differentiation				
Well	0.644 (0.443–0.937)	0.022	0.849 (0.558–1.291)	0.443
Moderately	Reference		Reference	
Poorly/undifferentiated	1.475 (1.104–1.972)	0.009	1.384 (1.017–1.885)	0.039
The extent of tumor				
Localized	Reference		Reference	
Regional	2.236 (1.666–3.000)	< 0.001	1.310 (0.839–2.044)	0.235
Distant	3.966 (2.335–6.736)	< 0.001	0.716 (0.144–3.560)	0.683
AJCC staging				
I	Reference		Reference	
II	1.952 (1.452–2.624)	< 0.001	0.958 (0.213–4.312)	0.956
III	2.942 (2.112–4.097)	< 0.001	2.000 (0.407–9.815)	0.393
IV	5.218 (3.066–8.885)	< 0.001	1.527 (0.183–12.747)	0.696
T staging				
T1	Reference		Reference	
T2	2.024 (1.517–2.699)	< 0.001	1.294 (0.284–5.884)	0.739
T3	2.561 (1.795–3.654)	< 0.001	0.698 (0.153–3.193)	0.643
T4	5.785 (3.494–9.579)	< 0.001	1.449 (0.270–7.785)	0.665
N staging				
N0	Reference			
N1	2.130 (0.877–5.170)	0.095		
M staging				
M0	Reference		Reference	0.176
M1	4.947 (2.814–8.694)	< 0.001	5.000 (0.485–51.541)	
Tumor size				
< 3 cm	0.912 (0.607–1.370)	0.657	0.991 (0.653–1.503)	0.965
3–5 cm	Reference		Reference	
5–10 cm	1.398 (0.997–1.960)	0.052	1.340 (0.931–1.927)	0.115
≥ 10	2.349 (1.621–3.402)	< 0.001	1.812 (1.179–2.786)	0.007
AFP				
Positive	Reference		Reference	0.022
Normal	0.243 (0.139–0.424)	< 0.001	0.698 (0.514–0.950)	
Fibrosis				
Normal	Reference		Reference	0.008
Cirrhosis	1.341 (1.045–1.721)	0.021	1.473 (1.107–1.960)	
MVI				
No	Reference		Reference	0.105
Yes	1.921 (1.471–2.510)	< 0.001	1.447 (0.926–2.259)	
Radiotherapy				
No	Reference		Reference	0.165
Yes	1.819 (1.039–3.186)	0.038	1.557 (0.834–2.909)	
Chemotherapy				
No	Reference		Reference	0.444
Yes	1.621 (1.166–2.254)	0.004	1.163 (0.789–1.715)	

Other race: American Indian/AK Native, Asian/Pacific Islander

*AFP* the highest AFP test results documented in the medical record prior to treatment, *MVI* microvascular invasion

**Fig. 2:**
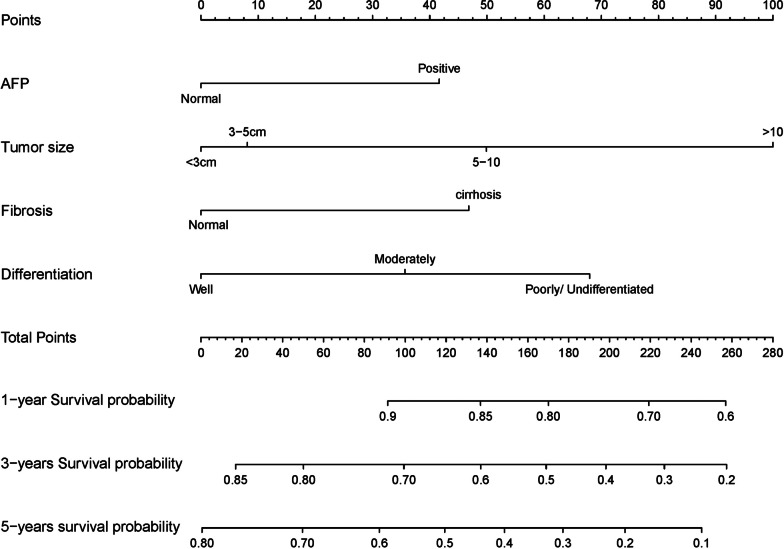
Nomogram predicting survival in HCC patients after partial hepatectomy

Based on the final multivariate Cox regression analysis, nomogram to predict prognosis of Hepatocellular carcinoma patients after surgery is shown in Fig. 2. The nomogram for predicting prognosis was developed based on the following 4 independent prognostic predictors: Differentiation (Well, Moderately, or Poorly/Undifferentiated), Tumor size (< 3 cm, 3–5 cm, 5–10 cm, or ≥ 10), AFP (Positive or Normal), and Fibrosis (Normal or Cirrhosis). For example, a patient with moderately differentiation, the tumor size is 4 cm, AFP is positive, and cirrhosis would have a total of 133 points (36 points for differentiation, 8 points for tumor size, 42 points for AFP, and 47 points for Fibrosis), the survival probability of 1-year, 3-year and 5-year is about 85%, 60%, and 45%, respectively.

### Nomogram performance in primary cohort

**Fig. 3:**
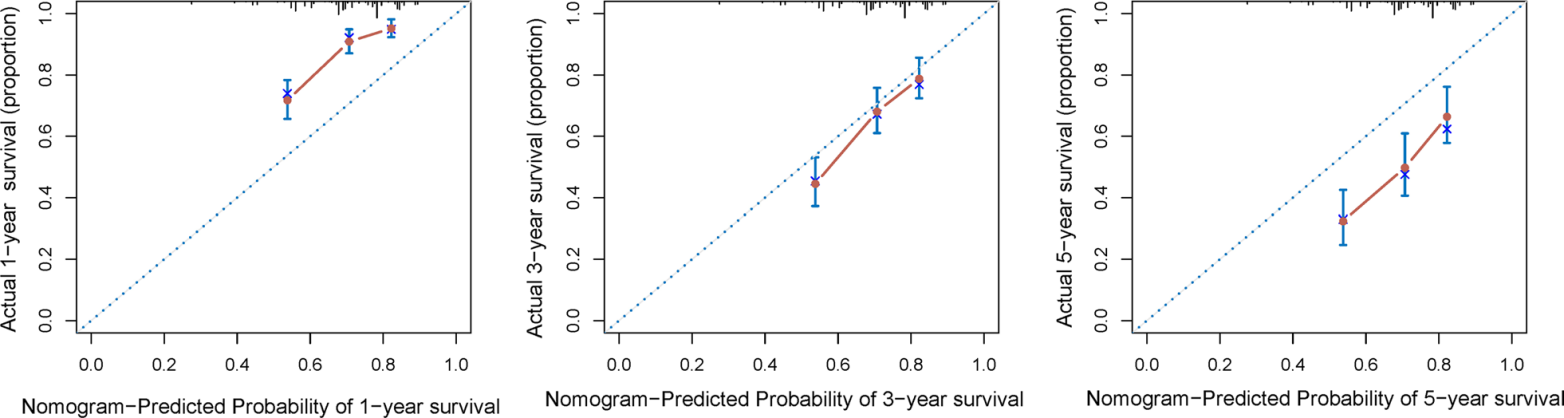
The calibration curve for 1-, 3- and 5-years survival probabilities in the primary cohort (B = 1000)

Calibration curves of the nomogram for 1-year, 3-year, and 5-year survival rate after surgery demonstrated a marked agreement between the prediction and actual observed outcomes in the primary cohort (Fig. 3). The C-indice for the primary cohort of the nomogram wax 0.705 (95% CI 0.669 to 0.742).

### External validation of the nomogram in the validation cohort

**Fig. 4:**
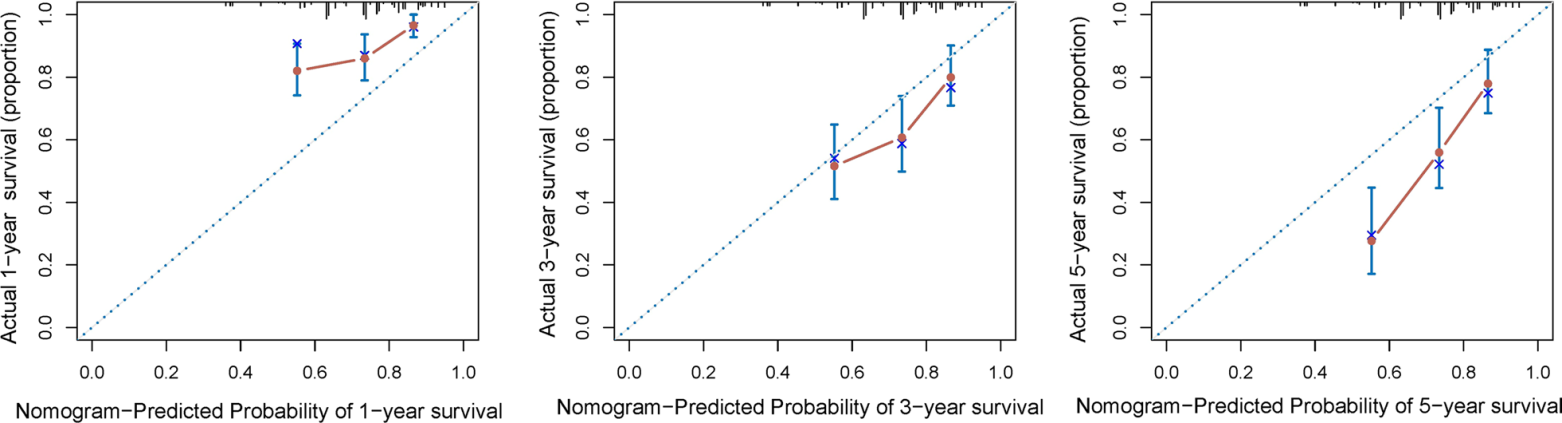
The calibration curve for 1-, 3- and 5-years survival probabilities in the validation cohort

Similar calibration for the survival probability of 1-year, 3-year, and 5-years survival after surgery was observed in the validation cohort (Fig. 4). In addition, the C-index of the nomogram for predicting prognosis was 0.681 (95% CI 0.625 to 0.737) for the validation cohort.

### Clinical use of the model

**Fig. 5:**
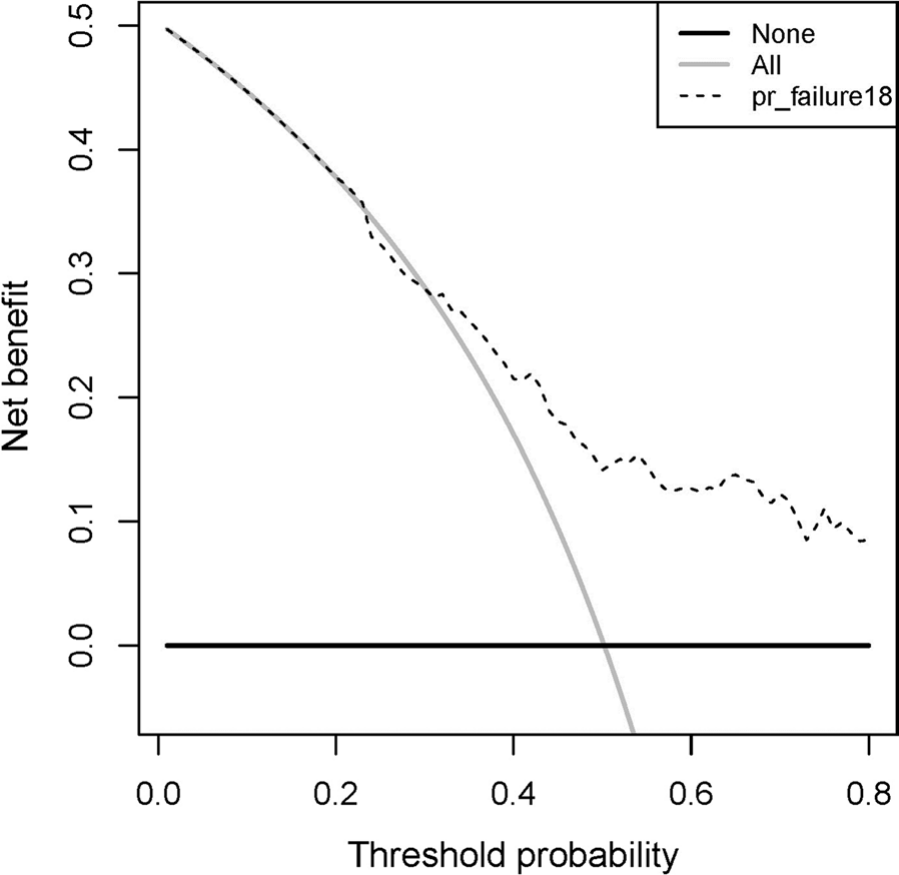
Decision curve analysis for the nomogram (B = 1000)

DCA was performed to assess the clinical utility of the nomogram in the primary cohort. The DCA results indicate our nomogram offered a net benefit at the wider range of threshold probability between 0.30 and 1 for predicting prognosis in the training cohort (Fig. 5).

## Discussion

Our study developed and validated a nomogram to predict the prognosis of hepatocellular carcinoma patients after partial hepatectomy individually. Our findings suggest that Differentiation, Tumor size, AFP, and Fibrosis are the most relevant predictors of prognosis. Importantly, the nomogram constructed based on the above four predictors can provide good discrimination and calibration for predicting the prognosis of HCC. Additionally, our nomogram has been externally validated, which indicated a high level of agreement between observed and predicted results. Based on our experience with this nomogram, we believe that it can be used for risk stratification and a personalized operating system to predict the prognosis of HCC patients. Clinical decision-makers can use this nomogram to plan individualized surgery, to determine follow-up interval monitoring, and to plan adjuvant therapy.

Additionally, one of the strengths of this study was the fact that it included a wide range of clinicopathological features previously reported to be associated with the prognosis of HCC patients after surgical resection [6, 21–23]. Despite a variety of factors that have been identified as associated with outcomes, there is a relative lack of consensus about what determines prognosis. According to some studies, male, blood transfusion, liver cirrhosis, larger tumor size than 5 cm, microvascular invasion, high serum alpha-fetoprotein, and satellite lesions are associated with worse outcomes [6, 24]. In contrast, other researchers have reported no correlation between microvascular invasion [25], sex [26, 27], tumor size < 10 cm [28], or perioperative blood transfusions [29] and long-term survival. Similarly, we found no association between sex, or microvascular invasion, and prognosis. In addition, we also included radiotherapy and chemotherapy in the analysis, which was not found in previous studies, but we did not observe statistical significance, which may be related to the insensitivity of HCC to radiotherapy and chemotherapy.

For patients such as those with HCC, the prognosis may be heterogeneous, so accurate risk stratification of the patients is critical [4, 30]. Complete surgical resection is the most effective treatment for patients with HCC, provides the best possibility of long-term survival [31–33]. However, postoperative tumor recurrence and liver failure caused by liver cirrhosis are still important factors affecting the prognosis of patients, which is also an important reason affecting postoperative adjuvant selection. Rather than using AJCC/BCLC staging data derived from large cohorts, patients could benefit from nomogram by providing them with more personalized prognostic information. Hu and Liu et al. [34, 35] previously proposed nomograms among patients with HCC based on the data of SEER database, including patients with early and late stages. Patients with metastasis should not be included, as their prognoses are likely to be greatly affected by unresectable distant metastatic lesions. Therefore, we chose to analyze only patients who are able to undergo radical resection, in order to provide prognostic information for patients who are most likely to receive HCC surgery. Furthermore, our proposed nomogram showed good discrimination and calibration, as evidenced by a C-index of 0.705 and calibration curve (Figs. 3 and 4).

It is important to understand that the nomogram is designed to interpret an individual's need for additional care or treatment. However, a single level of discrimination or degree of miscalibration in the risk-prediction performance, differentiation, or calibration, did not represent the clinical consequences [36]. Thus, to demonstrate clinical utility, we used decision curve analysis to evaluate whether our nomogram-assisted decision-making would improve the prognosis of patients. Based on threshold probability, decision curve analysis can provide insight into clinical consequences, from which the net benefit can be determined [37, 38]. Decision curve shows that when the threshold probability of nomogram is greater than 30%, doctors use the nomogram to predict the postoperative survival rate of HCC patients, and the disposal of patients according to the and the disposal of patients (such as making follow-up plans) according to the results can make patients obtain net benefits results can make patients obtain net benefits. Several limitations were present in this retrospective study. First, a limited number of predictive factors were available from SEER program data when this nomogram was developed, such as hepatitis B reactivation after chemoimmunotherapy, Child score of liver function or postoperative AFP level, etc. Second, postoperative adjuvant therapy was unknown (such as targeted therapy, immunotherapy or interventional therapy), we cannot consider the change in postoperative adjuvant therapy during the study; therefore, no assessment of the potential impact of postoperative adjuvant therapy on the nomogram can be made. However, there are inherent limitations in retrospective, population-based studies, and these drawbacks may raise doubts about the generalizability of the findings. Third, it is necessary to conduct additional external validations despite the present nomogram being validated internally and externally.

## Conclusion

We have developed and validated a low-cost and low-risk model nomogram using a large multicenter database to predict postoperative prognosis for HCC patients after partial hepatectomy. The proposed nomogram is highly predictive and has moderate calibration and discrimination, potentially contributing to the process of managing HCC patients after partial hepatectomy in an individualized way.

## Data Availability

The data analyzed during the current study are available from the corresponding author on reasonable request.

## Abbreviations

HCC: Hepatocellular carcinoma
DCA: Decision curve analysis
BCLC: Barcelona Clinic Liver Cancer
AJCC: American Joint Committee on Cancer
AFP: Alpha fetoprotein
MVI: Microvascular invasion
